# Experimental Investigation on Bending Properties of DP780 Dual-Phase Steel Strengthened by Hybrid Polymer Composite with Aramid and Carbon Fibers

**DOI:** 10.3390/polym16223160

**Published:** 2024-11-13

**Authors:** Jerzy Marszałek

**Affiliations:** Department of Mechanical Engineering Fundamentals, Faculty of Mechanical Engineering and Computer Science, University of Bielsko-Biala, Willowa 2, 43-309 Bielsko-Biala, Poland; jmarszalek@ubb.edu.pl

**Keywords:** metal/FRP hybrid structure, three-point bending test, effect of hybridization, weight reduction, mechanical properties

## Abstract

Lowering passenger vehicle weight is a major contributor to improving fuel consumption and reducing greenhouse gas emissions. One fundamental method to achieving lighter cars is to replace heavy materials with lighter ones while still ensuring the required strength, durability, and ride comfort. Currently, there is increasing interest in hybrid structures obtained through adhesive bonding of high-performance fiber-reinforced polymers (FRPs) to high-strength steel sheets. The high weight reduction potential of steel/FRP hybrid structures is obtained by the thickness reduction of the steel sheet with the use of a lightweight FRP. The result is a lighter structure, but it is challenging to retain the stiffness and load-carrying capacity of an unreduced-thickness steel sheet. This work investigates the bending properties of a non-reinforced DP780 steel sheet that has a thickness of 1.45 mm (S_1.45_) and a hybrid structure (S_1.15_/ACFRP), and its mechanical properties are examined. The proposed hybrid structure is composed of a DP780 steel sheet with a thickness of 1.15 mm (S_1.15_) and a hybrid composite (ACFRP) made from two plies of woven hybrid fabric of aramid and carbon fibers and an epoxy resin matrix. The hybridization effect of S_1.15_ with ACFRP is investigated, and the results are compared with those available in the literature. S_1.15_/ACFRP is only 5.71% heavier than S_1.15_, but its bending properties, including bending stiffness, maximum bending load capacity, and absorbed energy, are higher by 29.7, 49.8, and 41.2%, respectively. The results show that debonding at the interface between S_1.15_ and ACFRP is the primary mode of fracture in S_1.15_/ACFRP. Importantly, S_1.15_ is permanently deformed because it reaches its peak plastic strain. It is found that the reinforcement layers of ACFRP remain undamaged during the entire loading process. In the case of S_1.45_, typical ductile behavior and a two-stage bending response are observed. S_1.15_/ACFRP and S_1.45_ are also compared in terms of their weight and bending properties. It is observed that S_1.15_/ACFRP is 16.47% lighter than S_1.45_. However, the bending stiffness, maximum bending load capacity, and absorbed energy of S_1.15_/ACFRP remain 34.4, 11.5, and 21.1% lower compared to S_1.45_, respectively. Therefore, several modifications to the hybrid structure are suggested to improve its mechanical properties. The results of this study provide valuable conclusions and useful data to continue further research on the application of S_1.15_/ACFRP in the design of lightweight and durable thin-walled structures.

## 1. Introduction

Reducing greenhouse gas emissions such as carbon dioxide (CO_2_) and ensuring good air quality are major challenges facing humanity. In 2017, around 20% of total greenhouse gas emissions in the EU originated from road transport. Thus, increased emphasis has recently been placed on reducing greenhouse gas emissions in the passenger transport sector. Vehicle weight reduction is a well-known strategy for improving fuel consumption in combustion engines and, consequently, reducing CO_2_ emissions into the atmosphere. It is assumed that a 10% reduction in vehicle weight results in a 6–8% fuel economy improvement [1]. In the case of electric vehicles, a 10% weight reduction typically results in a 13.7% increase in the driving range based on a single battery charge [2]. The considerable potential for vehicle weight reduction is due to the body, which represents about 25% of the total mass of a vehicle [3,4]. This is because the vehicle body, especially that of mass-produced passenger vehicles, is usually made of steel, which is one of the heaviest construction materials. The vehicle body is the main load-carrying structure that ensure overall vehicle properties. More importantly, it ensures a high level of safety for drivers and passengers by ensuring appropriate strength, stiffness, and deformation work. Therefore, a significant weight reduction may result in low stiffness and strength of a vehicle body and an increase in noise and vibrations. For these reasons, vehicle body weight reduction, while maintaining other critical properties, is a complex and multifaceted engineering problem.

To meet these challenges, the automotive industry is increasing the use of advanced high-strength steels and ultra-high-strength steels that ensure lightweightness and quality of components while improving the safety of vehicles [5,6]. Thin-walled vehicle components made from such steels are lighter and meet key criteria, including high stiffness, high strength, and good energy absorption. Automotive steels are still under development to improve their mechanical properties and enable the design of lightweight and durable vehicle bodies [7,8,9]. In addition to continually developing automotive steels, there have been attempts to use other materials to manufacture lighter vehicle bodies. A promising approach in this context is the use of adhesively-bonded steel sheets and fiber-reinforced polymer (FRP) composites. Steel/FRP joints are hybrid structures [10] that combine the benefits of automotive steels and the high strength-to-weight ratio of FRP composites. A high weight-reduction potential of steel/FRP hybrid structures is obtained by reducing the thickness of steel sheets by using lightweight FRP composites to ensure necessary mechanical properties.

Some thin-walled parts of the vehicle body, such as B-pillars, doorsill beams, and side-door beams, are designed to carry bending loads from the transverse direction [11]. Their stiffness and strength are examined primarily using three-point or four-point bending tests [12,13]. According to Table 1, bending tests are also performed on steel/FRP hybrid structures, including both complex-shaped vehicle body parts and test specimens. Many research papers present examples of the use of carbon fiber-reinforced polymer (CFRP) composites to reinforce thin-walled steel structures without reducing the thickness of the sheet, which increases the weight of the structures, despite improving their mechanical properties. However, the literature contains few comparisons of the bending properties in terms of bending stiffness, maximum bending load capacity, and absorbed energy between steel sheets and hybrid structures composed of thinner steel sheets and FRP composites. It should be noted that experimental research on steel/FRP hybrid structures is still at the basic stage, which focuses on their mechanical properties, adhesive joint strength, and formability. In addition, the design of steel/FRP hybrid structures becomes more complicated if the FRP composite is fabricated by combining two or more different types of fibers. However, FRP composites made of one type of fiber and FRP hybrid composites, owing to their advantages and large design space, are widely applied in various fields of industry, such as aerospace, automotive, marine, and building construction [14,15,16,17]. A larger design space for FRP hybrid composites results from the possibility of combining the advantages of individual fibers. For example, more ductile glass or aramid fibers can be used to partially replace carbon fibers, creating higher strength and stiffness, which prevents brittle breakage of the composite during loading [18,19].

Table 1 shows that thin-walled steel structures in the automotive industry are mainly reinforced with CFRP composites because carbon fibers provide especially high stiffness, high load-bearing qualities, and low weight. As stated above, despite their many beneficial properties, carbon fibers are very brittle, which may result in premature delamination or fracture of CFRP composites. To improve the ultimate strain of CFRP composites, a portion of the carbon fibers can be replaced by more ductile aramid fibers. The density of aramid fibers (1.44 g/cm^3^) is lower than that of carbon (1.7 g/cm^3^) and glass (2.5 g/cm^3^) fibers, which provides greater potential weight reduction [28,29]. Despite the higher cost of aramid fibers compared with glass fibers, aramid fiber-reinforced polymer (AFRP) composites have better mechanical properties (i.e., tensile strength and elastic modulus), lower density, and improved impact properties (i.e., energy absorption capabilities), which is very important in the case of vehicle body components. For this reason, the automotive industry is particularly interested in AFRP and CFRP composites when designing lightweight and durable thin-walled components. Typical reinforcement forms containing a combination of aramid and carbon fibers are plain and twill weave hybrid fabrics. Although aramid fibers are widely used in the aerospace and automotive industries [30], no literature is available on the applications of aramid-carbon hybrid fabrics as a component of steel/FRP hybrid structures. Therefore, the objective of this research is the experimental investigation of the bending response of steel/FRP hybrid structure with reinforcement in the form of aramid and carbon fibers. Based on the above analysis, this study covers some important issues, including:

▪ Reducing the weight of a steel sheet by reducing its thickness and then strengthening it with aramid-carbon fiber-reinforced polymer (ACFRP) hybrid composite to obtain a steel/ACFRP hybrid structure, which is between 15% and 20% lighter;
▪ An investigation of the hybridization effect of a steel sheet and ACFRP by comparing their bending properties with a steel/ACFRP hybrid structure;
▪ A comparison of the experimentally-determined bending properties of unreduced-thickness steel sheet specimens and the steel/ACFRP hybrid structure specimens in terms of the bending stiffness, maximum bending load capacity, and absorbed energy;
▪ A comparison between the bending properties of the steel/ACFRP hybrid structure specimens and other hybrid structures available in the literature.

In view of the above, the outline of the paper is given as follows. In Section 2, the steel/ACFRP hybrid structure and its constituent components are characterized in detail. Moreover, the method used to fabricate the specimens and the procedure of the three-point bending tests are presented. The results of these tests are shown and discussed in Section 3. Finally, the main conclusions of the study are provided in Section 4.

## 2. Materials and Methods

### 2.1. Materials

**Figure 1:**
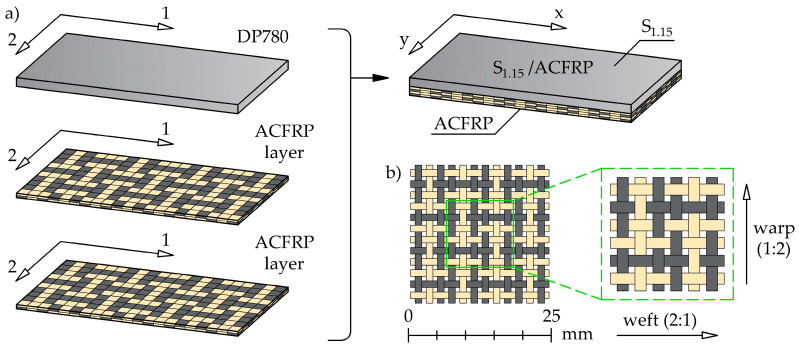
(**a**) Schematic illustration of the hybrid structure investigated in this study; (**b**) weave pattern in the hybrid aramid/carbon fiber fabric.

Figure 1a illustrates a schematic diagram of the S_1.15_/ACFRP hybrid structure and its stacking sequence. The hybrid structure was composed of an adhesively-bonded steel sheet with a thickness of 1.15 mm (designated as S_1.15_) and two-ply aramid-carbon fiber-reinforced polymer (ACFRP) hybrid composite. S_1.15_ was made from cold-rolled DP780 dual-phase steel, which is widely used in the automotive industry to manufacture complex-shaped vehicle body components due to its effective combination of strength and formability. Many researchers have experimentally evaluated the mechanical properties and chemical composition of DP780 steel [31,32,33]. The selected mechanical properties of DP780 steel are presented in Table 2. ACFRP consisted of a hybrid bidirectional fabric that is made of aramid and carbon fiber tows placed in a plain-weave pattern, as shown in Figure 1b. Detailed information about the fabric, aramid fibers, and carbon fibers is listed in Table 3. The fabric was not balanced because the aramid and carbon tows were present in a 1:2 ratio in the warp direction and a 2:1 ratio in the weft direction, respectively. The polymer matrix of ACFRP consisted of an epoxy resin (Biresin^®^ CR122) and a hardener (Biresin^®^ CH122-5), with a mixing ratio of 100:30. The same matrix was also used for bonding between S_1.15_ and ACFRP. The selected properties of the matrix available in the manufacturer’s data sheet are given in Table 4. As shown in Figure 1, the 1-2 in-plane coordinate system used the coordinates of the constituent components. The 1-axis indicates the rolling direction of S_1.15_ and the weft direction of the hybrid fabric. The x- and y-axes denote the in-plane longitudinal and transverse directions, respectively, in the global coordinate system of S_1.15_/ACFRP. Seven aramid tows and three carbon tows were symmetrically arranged with respect to the *x*-axis. All materials used in this study were supplied by local suppliers.

### 2.2. Fabrication of Specimens

**Figure 2:**
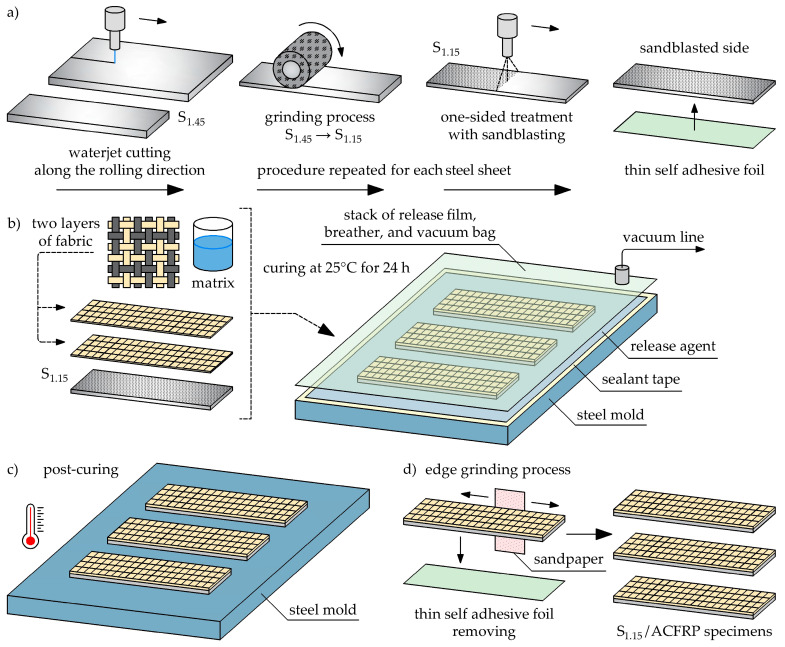
Fabrication process of the S_1.15_/ACFRP specimens: (**a**) preparation of the S_1.15_ steel sheets; (**b**) vacuum bag process; (**c**) post-curing process; (**d**) edge grinding process and final shape of the hybrid specimens.

**Figure 3:**
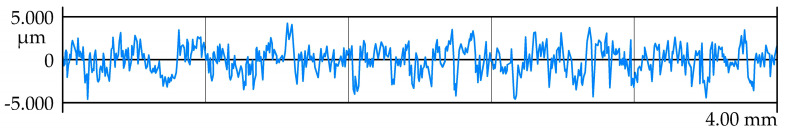
Surface roughness profile of the S_1.15_ steel sheet after sandblasting.

The detailed fabrication procedure of the S_1.15_/ACFRP specimens using a vacuum bagging technique is shown schematically in Figure 2. First, an external release agent was applied to the mold surface at room temperature to ensure the easy removal of the specimens after curing. Before this, the surface of the mold was thoroughly cleaned and then left at room temperature for two hours to complete drying. Thereafter, three steel sheets with dimensions of 120 mm × 20 mm were cut along the rolling direction from a DP780 steel plate with a thickness of 1.45 mm using a waterjet method. Using a precision grinding machine, the thickness of these steel sheets was reduced to 1.15 mm. One side of each steel sheet was treated with sandblasting to improve its surface roughness and achieve an enhanced bonding quality with ACFRP. The steel sheets were washed with warm water and degreased with solvent to wipe off any surface contaminants and metal scraps. Finally, the steel sheets were left to dry. The second surface of each steel sheet was sealed with a thin self-adhesive foil to protect it from contact with the matrix during the manufacturing process. Figure 3 shows the typical surface roughness Ra (in units of μm) of each steel sheet. The roughness profiles reported here were measured along the rolling direction at a measuring length of 4 mm using a profilometer. The epoxy matrix system was prepared by mixing an epoxy resin and hardener in a weight ratio of 100:30 with a mechanical mixer at 300 rpm for two minutes. Then, the matrix system was heated from ambient temperature to 28 °C to slightly reduce its viscosity and facilitate the rise of air bubbles to the surface. The air bubbles were completely removed from the matrix system using a laboratory spoon.

The stacking process was started by placing three steel sheets on the mold plate. Before placing the first hybrid fabric layer, a thin layer of matrix was applied on the sandblasted surface of each steel sheet. One layer of hybrid fabric impregnated with the matrix was then applied to each matrix-coated steel sheet, and a plastic roller was used to press the fabric against the steel sheet surface to remove excess matrix and air bubbles. Next, the matrix was reapplied to the surface of the hybrid fabric, and the second layer of the hybrid fabric was placed. Afterward, a final epoxy resin coating was applied, and the plastic roller was used once again to remove the air bubbles. The entire setup was covered by a vacuum bag sealed with sealant tape. The vacuum bag remained attached to the vacuum pump to remove bubbles from the prepared specimens. The specimens were left to cure at room temperature for 24 h under vacuum and later extracted and subjected to post-curing according to the following cycle: 1 °C/min ramp to 80 °C, 5 h isothermal hold at 80 °C, and 1 °C/min cool to room temperature. Finally, the self-adhesive foil was removed from the steel sheets, and the edges of each specimen were carefully sanded with sandpaper to remove excess matrix and obtain a finished surface.

**Figure 4:**
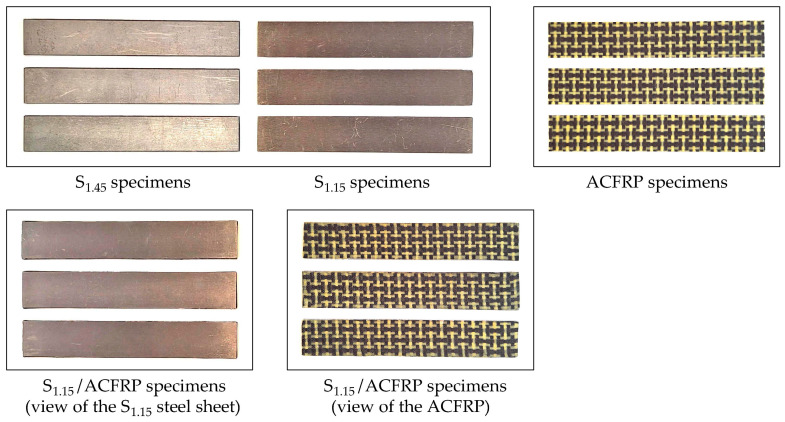
Photographs of the prepared specimens.

As mentioned above, this study also examined the bending behavior of S_1.45_ and the constituent components of the S_1.15_/ACFRP hybrid structure. The S_1.45_ specimens were cut along the rolling direction from a DP780 steel plate with a thickness of 1.45 mm using the waterjet method. Three specimens were subjected to grinding to obtain the S_1.15_ specimens with a thickness of 1.15 mm. Two-ply ACFRP specimens were constructed using exactly the same procedure as the S_1.15_/ACFRP specimens. The length and width of all of the specimens were equal to 120 mm and 20 mm, respectively. Optical images of all of the prepared specimens are displayed in Figure 4, and their average properties are listed in Table 5.

### 2.3. Three-Point Bending Tests

**Figure 5:**
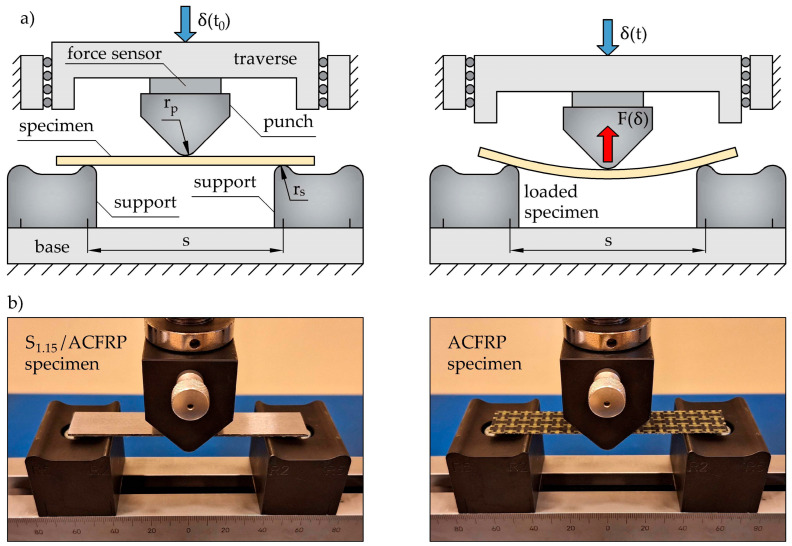
(**a**) Three-point bending test setup; (**b**) photographs of the bend fixture and exemplary specimen during the test.

The particularly important mechanical properties of steel/FRP hybrid structures intended for the automotive industry are bending stiffness and strength. This is because automotive components are generally thin-walled, and bending is the main loading condition. The bending behaviors of S_1.15_/ACFRP, its constituent components (i.e., S_1.15_ and ACFRP), and S_1.45_ were studied by use of a three-point bending test (Figure 5a). To comparatively analyze the bending properties of the specimens, all of the tests were performed under the same conditions, including span length (s = 80 mm), support radius (r_s_ = 2 mm), punch radius (r_p_ = 5 mm), constant punch displacement rate (1 mm/min), temperature (23 ± 2 °C), and relative humidity (50 ± 10%). The data obtained from the three-point bending tests included the deflection δ of the specimens at the midspan (equivalent to the punch displacement) and the load F (equivalent to the reaction force generated by specimens and experienced by a force sensor). These values were recorded by data acquisition software and used to plot the load–deflection curves F(δ) for further analysis. At the initial time t_0_, the punch was in contact with the specimens, and the deflection was equal to zero (during the tests, the deflection increased linearly with time t). The tests were carried out using a universal testing machine (Inspekt Table Blue 5 kN, Hegewald & Peschke, Nossen, Germany). Figure 5b provides a photograph of the exemplary test specimen under loading. In the case of the S_1.15_/ACFRP specimens, a bending load was applied on the S_1.15_ surface, which means that S_1.15_ and ACFRP were exposed to compressive and tensile loads, respectively.

**Figure 6:**
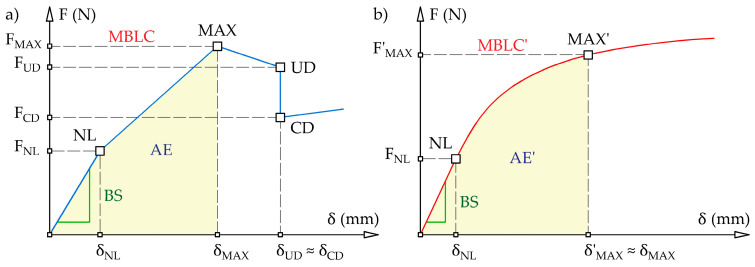
Types of load–deflection curves presented in this study: (**a**) five-stage bending response of the metal-FRP hybrid structure specimen; (**b**) two-stage bending response of the high ductility steel sheet and thin FRP specimens.

In order to analyze the bending behaviors of the specimens, some related parameters such as bending stiffness (BS), maximum bending load capacity (MBLC), and absorbed energy (AE) were determined based on the experimental data. According to preliminary studies, the hybrid structure (i.e., steel/FRP) and its constituent components (steel and FRP) may have had fundamentally different load–deflection responses. Under a three-point bending load, the hybrid structure specimens may have exhibited a five-stage response, as shown schematically in Figure 6a. The non-linear load–deflection response of the hybrid structure is presented in a simplified way using a multi-linear curve that is defined by a set of points designated as NL, MAX, UD, and CD. The load initially creates a linear increase. The non-linear point (NL) indicates the change in the initial linear section of the load–deflection curve into a non-linear form, which occurs at the deflection δ_NL_ and the corresponding load F_NL_ owing to the beginning of plastic deformation of the steel sheet. The non-linear part of the curve increases until the maximum point (MAX) is reached. The maximum bending load capacity (MBLC), equivalent to the peak load F_MAX_, occurs at this point, and the corresponding deflection is δ_MAX_. Between the NL and MAX points, an initiation of different progressive failure mechanisms (i.e., debonding, delamination, or fiber breakage) can be observed. After point MAX, the bending load capacity is decreased, indicating unstable debonding growth along the interface between the steel sheet and the FRP composite. Adhesive bonding between the steel sheet and FRP composite reaches its ultimate strength at the ultimate deflection point (UD), with coordinates of deflection δ_UD_ and load F_UD_. This results in complete debonding failure and a rapid load drop to the complete debonding point (CD) at the deflection δ_CD_ and the corresponding load F_CD_. Despite this, the hybrid specimen retains a certain load-bearing capacity, and the bending test is continued until the specimen fractures or when the reliability of the test begins to decrease due to a large deflection.

According to Figure 6a, the bending stiffness (BS) is equal to the slope within the linear-elastic region of the load–deflection curve. The bending stiffness is an important mechanical property of a material or structure that reflects the ability to resist deformation. Additionally, the absorbed energy (AE), equivalent to the work done by the bending force, is calculated as the area under the load–deflection curve (in this study, the trapezoidal integration rule was used for this purpose) and considering the deflection corresponding to δ_MAX_.

The bending response of the constituent components of the hybrid structure (i.e., steel and FRP) is shown in Figure 6b. The high ductility and strain hardening capacity of many automotive steels and the low bending stiffness of thin FRPs (those made of several layers) cause their bending response to be divided into only linear and non-linear stages. In this case, just the NL point with coordinates of deflection δ_NL_ and load F_NL_ may be identified because this kind of specimen usually shows significantly higher deflection with no damage in the measurement range. In addition, sliding behavior of the specimens on the supports occurs upon further increasing the deflection, which decreases the accuracy of the load–deflection curves. In this situation, the true value of the maximum bending load capacity and absorbed energy of steel and FRP specimens cannot be determined. Therefore, the load capacity and absorbed energy of the hybrid structure and its constituent components can be compared for one fixed value of deflection. It is the deflection δ_MAX_ at which the hybrid structure specimens achieve their maximum bending load capacity. For the steel and FRP specimens, the apparent point MAX’ with deflection δ’_MAX_ and corresponding load F’_MAX_ is marked in Figure 6b, where the deflection δ’_MAX_ is equal to the deflection δ_MAX_ of the hybrid specimens. Then, the apparent maximum bending load capacity (MBLC’) and absorbed energy (AE’) of the steel and FRP specimens can be determined using apparent point MAX’. Based on the linear-elastic stage of the load–deflection curve, the only true parameter that can be determined for the steel and FRP specimens is bending stiffness (BS).

## 3. Results and Discussion

### 3.1. Bending Response of the S_1.15_/ACFRP Specimens

**Figure 7:**
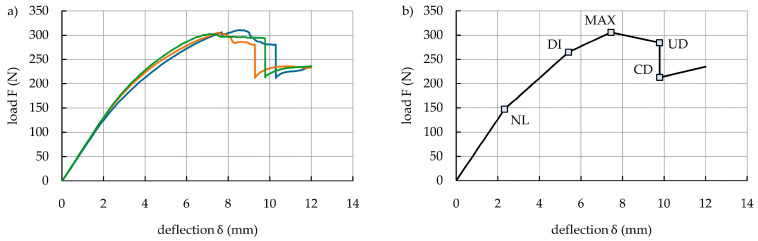
(**a**) Load–deflection curves of the three S_1.15_/ACFRP specimens; (**b**) average load–deflection curve with characteristic points for all the S_1.15_/ACFRP specimens.

The load–deflection curves from the three tested S_1.15_/ACFRP specimens are presented in Figure 7a. As expected, the curves indicated five main loading stages during the tests. The boundaries of these stages in the form of characteristic points (i.e., NL, MAX, UD, and CD) are marked in the average curve (Figure 7b), and the corresponding deflection and load values are listed in Table 6. The results revealed good agreement during repeated tests. In the first stage (0-NL), the load and deflection maintained an approximately linear relationship because S_1.15_ and ACFRP both underwent elastic deformation. Additionally, no damage patterns appeared in the specimens during this stage. In the second stage (NL-MAX), the slope of the curves gradually decreased upon increasing deflection, indicating that the bending stiffness of the specimens slightly decreased due to local plastic deformation of S_1.15_. Between the NL and MAX points, an additional point DI is marked (the damage initiation point), with coordinates of deflection δ_DI_ and load F_DI_. At this point, cracking sounds with growing intensity began to appear, which meant the initiation of the progressive damage process of the hybrid structure. In the third stage (MAX-UD), the slope of the curves changed significantly.

Visual observations did not indicate any external damage, but cracking sounds with growing intensity occurred due to internal damage in the S_1.15_/ACFRP specimens. At the point UD, adhesive bonding between S_1.15_ and ACFRP reached its ultimate strength, which resulted in complete debonding failure and a rapid load drop to point CD (i.e., the fourth stage UD-CD). Then, the bending load was mainly carried by S_1.15_ (the fifth stage), and the specimens retained a certain bending stiffness and load-bearing capacity upon further increasing the deflection. The bending stiffness, maximum bending load capacity, and absorbed energy of the S_1.15_/ACFRP specimens under a bending load are summarized in Table 7.

**Figure 8:**
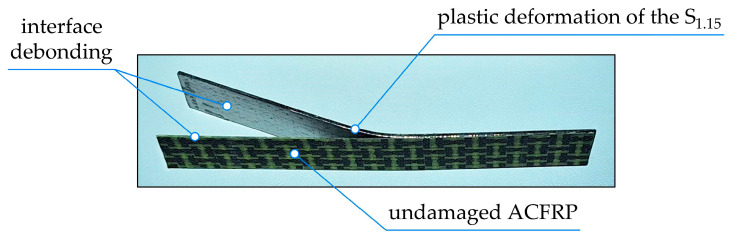
Macrograph of the S_1.15_/ACFRP specimen after the test.

After the tests were completed, the S_1.15_/ACFRP specimens were removed from the supports to observe their damage patterns. Figure 8 shows the image of the front side of the exemplary specimen, although all of the hybrid specimens experienced the same fracture mechanism. The primary mode of fracture in S_1.15_/ACFRP under a three-point bending load was debonding at the interface between S_1.15_ and ACFRP. S_1.15_ was permanently deformed in all of the tested specimens because it reached its peak plastic strain at the midspan, and there were no obvious cracks or fractures in S_1.15_ after the bending process. In turn, ACFRP in each tested specimen showed elastic behavior over the entire loading range because, in contrast to S_1.15_, its longitudinal edge remained a straight line. Additionally, there were no damage patterns in ACFRP, such as matrix cracks, breakage of longitudinal fiber tows, or the cracking of transverse fiber tows. This means that debonding at the interface between S_1.15_ and ACFRP was one of the main failure modes in S_1.15_/ACFRP when subjected to bending.

### 3.2. Bending Response of the S_1.45_ and S_1.15_ Specimens

**Figure 9:**
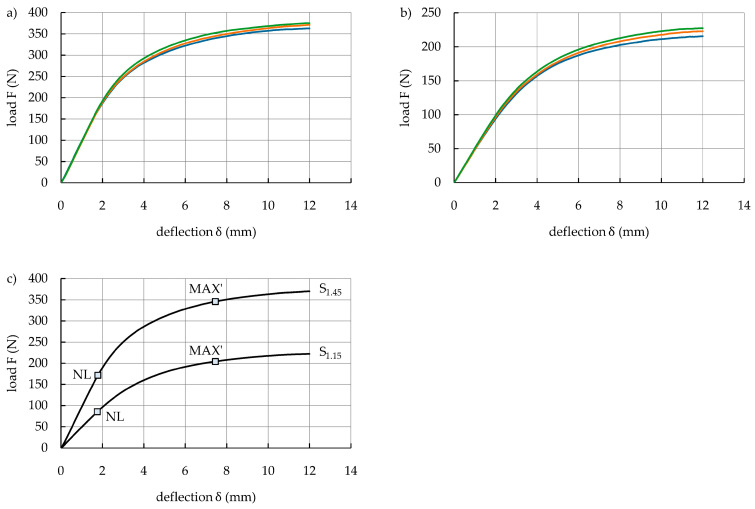
Load–deflection curves of DP780-grade steel sheets: (**a**) S_1.45_ specimens; (**b**) S_1.15_ specimens; (**c**) average load–deflection curves of the S_1.45_ and S_1.15_ specimens.

The load–deflection curves of the S_1.45_ and S_1.15_ specimens are shown in Figure 9a,b, respectively, in which the specimens display typical ductile behavior and a two-stage bending response. A comparison of the average load–deflection curves of these specimens is presented in Figure 9c, and the boundary of the linear and non-linear stages is marked in the form of the NL point. The tests showed good repeatability of the results. In the first stage (0-NL), the load increased linearly, and the specimens exhibited elastic deformation, which meant that they completely recovered when the load was released. In the non-linear stage (above NL), the slope of the curves decreased, indicating that plastic deformation was locally concentrated in the top and bottom surfaces of the specimens. Sliding behavior of the specimens on the supports occurred upon further increasing the deflection, which decreased the accuracy of the load–deflection curves. The ductility of DP780 steel and the sliding behavior between the specimens and supports caused the specimens to deform without fracture until the end of the test. Therefore, the true value of the maximum bending load capacity and absorbed energy of the steel specimens could not be determined. In this situation, the apparent maximum bending load capacity MBLC’ and absorbed energy AE’ were determined based on the apparent point MAX’, with characteristic deflection δ’_MAX_ and load F’_MAX_ (Figure 9c). The deflection δ’_MAX_ was equal to the deflection δ_MAX_ of the hybrid specimens (δ’_MAX_ = δ_MAX_ = 7.45 mm). The average bending results and mechanical properties of the S_1.45_ and S_1.15_ specimens under a bending loading are summarized in Table 8. As expected, all of the properties (i.e., bending stiffness, apparent maximum bending load capacity, and apparent absorbed energy) of the S_1.45_ specimens were higher than those of the S_1.15_ specimens. The bending stiffness of the S_1.45_ specimens was two times higher because their second moment of area was also two times higher than that of the S_1.15_ specimens.

### 3.3. Bending Response of the ACFRP Specimens

**Figure 10:**
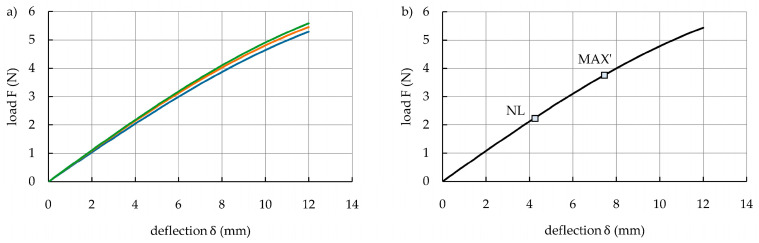
(**a**) Load–deflection curves of the three ACFRP specimens; (**b**) average load–deflection curve with characteristic points for all the ACFRP specimens.

The typical three-point bending load–deflection curves of three ACFRP specimens are presented in Figure 10a, which showed good repeatability. The bending process of these specimens was provided in both linear and non-linear stages. The boundary of these stages, in the form of the characteristic point NL, is marked in the average curve (Figure 10b). Owing to their small bending stiffness, the ACFRP specimens showed a large deflection during loading but no visible or audible effects over the whole measuring range that indicated damage. Furthermore, the specimens recovered to their original shape and initial position after unloading, indicating that they experienced only elastic strain. Their non-linearity above the NL point was related to their sliding effect on the supports. Taking this into account, the only reliable parameter for the ACFRP specimens obtained from the tests was the bending stiffness. However, for comparison purposes and further analyses, the apparent maximum bending load capacity MBLC’ and absorbed energy AE’ of the ACFRP specimens were determined based on the apparent point MAX’, with characteristic deflection δ’_MAX_ and load F’_MAX_ (Figure 10b), where δ’_MAX_ of the ACFRP specimens was equal to δ_MAX_ of the hybrid specimens (δ’_MAX_ = δ_MAX_ = 7.45 mm). The average bending results and mechanical properties of the ACFRP specimens under a bending loading are summarized in Table 9.

### 3.4. Hybridization Effect of S_1.15_ with ACFRP

**Figure 11:**
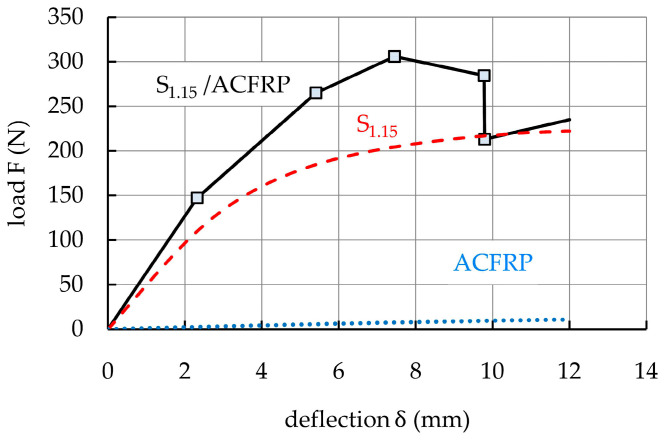
Comparison of the idealized load–deflection responses of S_1.15_/ACFRP, S_1.15_, and ACFRP specimens.

Figure 11 compares the average load–deflection curves of three types of specimens, including S_1.15_/ACFRP and its constituent components, i.e., S_1.15_ and ACFRP. As indicated in Section 2.3, based on the NL, DI, MAX, UD, and CD points, an idealized multi-linear curve was adopted in this study to represent the bending response of the S_1.15_/ACFRP specimens. For the S_1.15_ and ACFRP specimens, the average bending response curves were plotted with points NL and MAX’.

**Figure 12:**
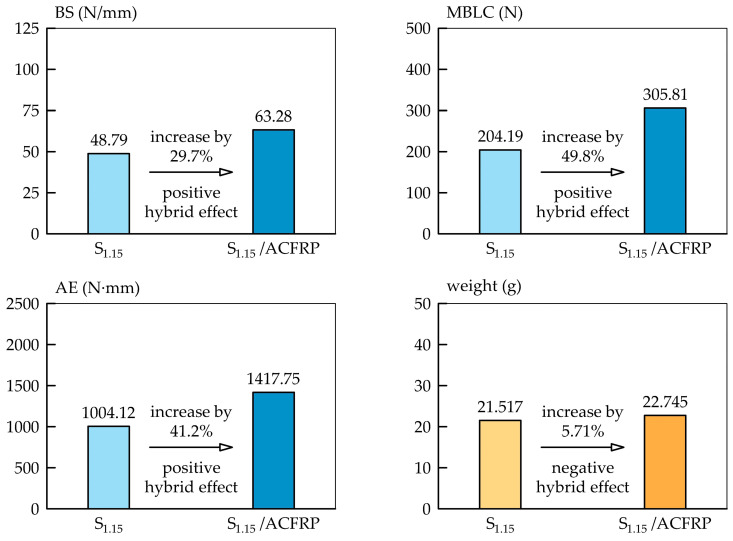
Bar graphs that provide a visual comparison of the bending stiffness, maximum bending load capacity, and absorbed energy of the S_1.15_/ACFRP and S_1.15_ specimens.

The bending properties of the ACFRP specimens in terms of bending stiffness, maximum bending load capacity, and absorbed energy were many times lower than those of the S_1.15_/ACFRP and S_1.15_ specimens. However, assembling S_1.15_ and ACFRP by using adhesive bonding provided many advantages with regard to bending properties. As shown in Figure 12, the bending stiffness of the S_1.15_/ACFRP specimens was 29.7% higher than that of the S_1.15_ specimens due to the greater thickness of S_1.15_/ACFRP and, consequently, a higher second moment of area. The S_1.15_/ACFRP specimens also showed a maximum bending load capacity, which was 49.8% higher than that of the S_1.15_ specimens. It was concluded that the ductile aramid fibers prevented premature failure of the more brittle carbon fibers, which in turn helped enhance the maximum bending load. The improvement in the maximum bending load capacity was also due to the high bending stiffness. Therefore, the thickness of ACFRP determined both the stiffness and bending load capacity of the S_1.15_/ACFRP specimens. Another benefit of the higher bending stiffness and maximum bending load capacity was the improved energy absorption performance of the S_1.15_/ACFRP specimens, which was 41.2% higher than that of the S_1.15_ specimens. The assembly of S_1.15_ and ACFRP by adhesive bonding significantly improved the bending stiffness, maximum bending load capacity, and absorbed energy compared to S_1.15_, which was attributed to hybridization. Importantly, the S_1.15_/ACFRP specimens retained a certain bending stiffness and could carry a load despite debonding. As shown in Figure 11, the load capacity of the S_1.15_/ACFRP specimens was slightly higher than that of the S_1.15_ specimens.

The above-mentioned advantages of the hybrid structure were achieved with a slight increase in weight compared to a steel sheet (the weight of S_1.15_/ACFRP was only 5.71% greater than that of S_1.15_).

### 3.5. Comparison of S_1.15_/ACFRP and S_1.45_

**Figure 13:**
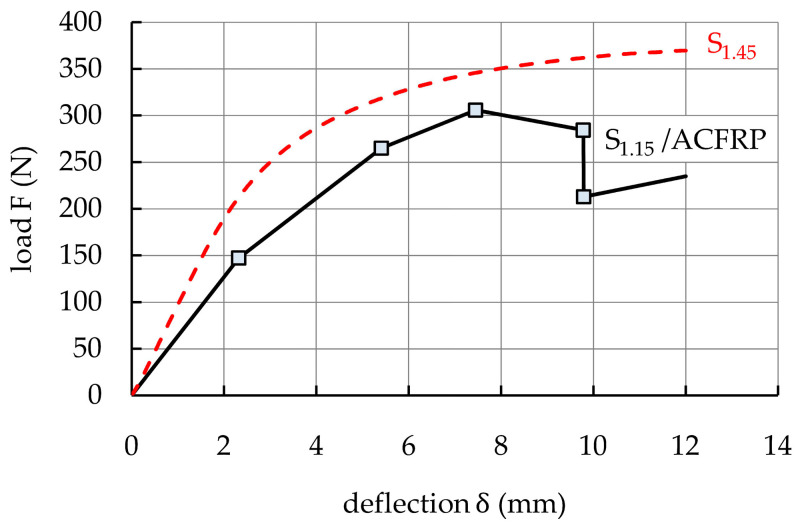
Comparison of the idealized load–deflection responses of the S_1.15_/ACFRP and S_1.45_ specimens.

**Figure 14:**
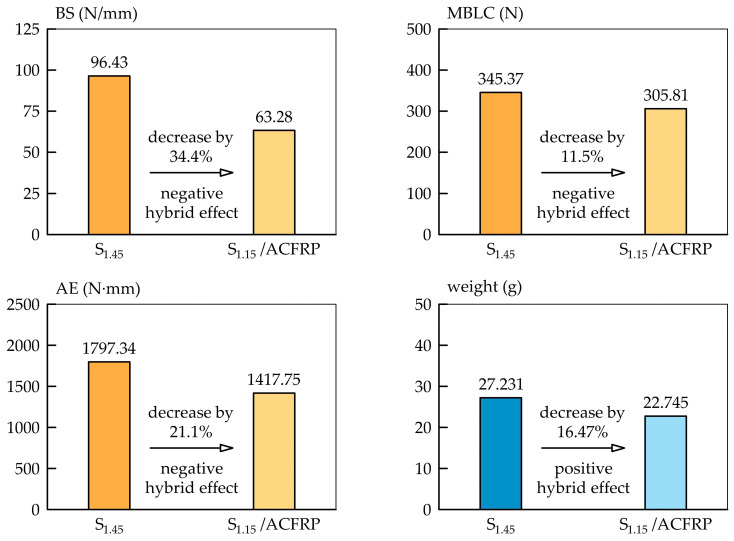
Bar graphs that provide a visual comparison of the bending stiffness, maximum bending load capacity, and absorbed energy of the S_1.15_/ACFRP and S_1.45_ specimens.

Figure 13 compares the average load–deflection curves of the S_1.15_/ACFRP and S_1.45_ specimens. Similar to Figure 11, the bending response of the S_1.15_/ACFRP specimens was idealized using a multi-linear curve. For the S_1.45_ specimens, the average bending response curve was plotted through the points NL and MAX’. Figure 14 graphically compares the bending performance parameters of the specimens. The S_1.15_/ACFRP specimens exhibited an initial bending stiffness that was 34.4% lower than that of S_1.45_. This was mainly due to the lower longitudinal modulus of the ACFRP hybrid composite compared to that of DP780 steel, resulting in lower bending stiffness in the initial phase of the loading. As is well known, the bending stiffness of a beam depends on both the elastic modulus of the material and the second moment of area related to the shape of the beam’s cross-section. For prismatic rectangular cross-sectional beams, the bending stiffness is mainly dependent on the thickness of the beam. Because the elastic modulus of the fabric-reinforced polymer composites is lower than that of steel sheets, it can be assumed that the thickness of the S_1.15_/ACFRP hybrid structure was not sufficient to make its bending stiffness higher compared to that of the S_1.45_ steel sheet. For the S_1.15_/ACFRP specimens, the maximum bending load capacity was 11.5% lower than that of the S_1.45_ specimens. This difference was due to the insufficient ultimate strength of the adhesive bonding between S_1.15_ and ACFRP. After debonding, the bending load was mainly carried by S_1.15_, and the bending load capacity of the S_1.15_/ACFRP specimens was approximately 40% lower than that of the S_1.45_ specimens. This was related to the greater thickness of S_1.45_. Finally, the S_1.15_/ACFRP specimens displayed absorbed energy, which was 21.1% lower than that of S_1.45_ due to their smaller bending load capacity over almost the whole measurement range.

### 3.6. Comparison of S_1.15_/ACFRP with Other Steel/FRP Hybrid Structures

The reinforcing effect of S_1.15_ with ACFRP in this paper was compared with similar hybrid structures in previous works. A comparative study was conducted on the bending stiffness, maximum bending load capacity, and weight increment for five hybrid structures, as shown in Table 10. Regardless of the type of reinforcement, the adhesive-bonded connection of steel and FRP significantly increased the bending stiffness and maximum bending load capacity, with a slight increase in weight. This was because the thickness of steel/FRP was greater than that of the non-reinforced steel sheet, which resulted in a higher second moment of area. Among the hybrid structures listed in Table 10, the greatest strengthening effect was shown by steel/GFRP/CFRP because, in this case, the steel sheet was reinforced with one layer of bidirectional glass fiber-reinforced polymer (GFRP) and three-ply unidirectional CFRP (the remaining steel sheets were reinforced with one-ply FRPs). The use of a large number of reinforcing plies also increased the weight of steel/GFRP/CFRP compared with the other hybrid structures, which were unidirectionally reinforced. Therefore, the tensile strength of the fibers was fully utilized in these structures during bending. In general, the proposed S_1.15_/ACFRP hybrid structure showed good bending properties, despite its bidirectional reinforcement.

### 3.7. Proposed Modifications of the S_1.15_/ACFRP Hybrid Structure

**Figure 15:**
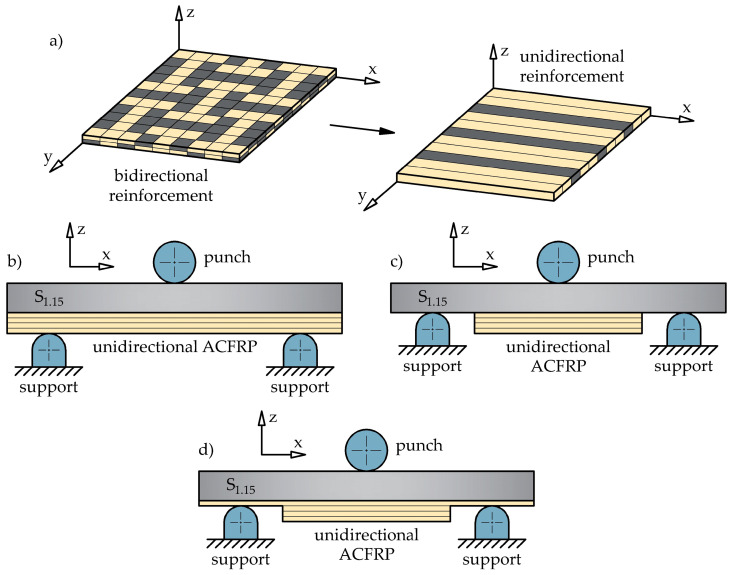
Methods for enhancing the bending properties of the S_1.15_/ACFRP hybrid structure: (**a**) replacement of bidirectional reinforcement with unidirectional reinforcement; (**b**) steel sheet strengthened with unidirectional ACFRP along the entire length; (**c**) steel sheet strengthened locally with unidirectional ACFRP; (**d**) application of additional local reinforcement in the S_1.15_/ACFRP hybrid structure.

According to Figure 14, the S_1.15_/ACFRP specimens exhibited lower bending properties than the S_1.45_ specimens. Therefore, the S_1.15_/ACFRP hybrid structure cannot effectively replace the DP780 steel sheet with a thickness of 1.45 mm in engineering applications. For this reason, it is suggested to replace bidirectional reinforcement fabric with unidirectional reinforcement, as shown in Figure 15a. Three strategies are proposed to improve the mechanical properties of the S_1.15_/ACFRP specimens without increasing their weight:

(1) Replace two layers of bidirectional reinforcement fabric with four layers of unidirectional reinforcement, as shown in Figure 15b. Aramid and carbon fibers arranged in the transverse direction do not carry the load during bending, and their potential for increased bending stiffness is not fully utilized in the S_1.15_/ACFRP specimens. In a four-ply unidirectional ACFRP composite, twice the amount of fibers are under tensile forces, which may translate into improved mechanical properties of the hybrid structure. The advantage of this approach is the same weight reduction since the four layers of the unidirectional reinforcement weigh approximately the same as the two layers of the bidirectional reinforcement fabric.
(2) Strengthen the S_1.15_ specimen with ACFRP composite locally in the region of the highest strain. According to Figure 15c, the three-point bend specimen should be strengthened in the tension zone at the midspan due to the maximum bending moment. An additional advantage of this approach is a greater reduction in the weight of the hybrid structure.
(3) Applying an ACFRP composite, consisting of a combination of a layer along the entire length of the S_1.15_ specimen and additional layers at the midspan, provides a local reinforcement effect, as shown in Figure 15d. The main purpose of using the first layer is to enhance the surface adhesion between S_1.15_ and ACFRP and, thus, increase the strength of the connection. This is important because the adhesive bond between the steel and polymer composite is usually less durable than the bond between the composite layers.

The advantages of the S_1.15_/ACFRP hybrid structure investigated in this study were not fully realized due to premature debonding between S_1.15_ and ACFRP. Therefore, improving the interfacial adhesion between S_1.15_ and ACFRP is the key to preparing a hybrid structure with high performance because then the load can be more effectively transferred from S_1.15_ to ACFRP. Investigating the above-mentioned approaches for improving the bending properties of the hybrid structure must be carried out while improving the quality of the interfacial adhesion between joined surfaces.

## 4. Conclusions

In this work, experimental studies were carried out to investigate the bending properties of the S_1.15_/ACFRP hybrid structure and its potential for designing lighter, thin-walled structures. For this purpose, four types of flat rectangular specimens, i.e., S_1.15_/ACFRP, DP780-grade steel sheet with a thickness of 1.15 mm (S_1.15_), DP780-grade steel sheet with a thickness of 1.45 mm (S_1.45_), and two-ply aramid-carbon fiber-reinforced polymer hybrid composite (ACFRP), were subjected to three-point bending tests. Their bending stiffness, maximum bending load capacity, absorbed energy, and weight were compared. The most important findings of the study are summarized as follows:

(1) Reducing the thickness of the steel sheet from 1.45 mm to 1.15 mm decreased its weight by approximately 20.98%, but assembling S_1.15_ and ACFRP by adhesive bonding obtained an S_1.15_/ACFRP hybrid structure with a thickness of 1.58 mm, which was 16.47% lighter than S_1.45_ (i.e., effective weight reduction was achieved).
(2) Due to hybridization, S_1.15_/ACFRP exhibited improved bending properties compared to S_1.15_ in terms of bending stiffness, maximum bending load capacity, and absorbed energy, with increases of 29.7, 49.8, and 41.2%, respectively. Its weight was only 5.71% greater than that of S_1.15_.
(3) The results showed that debonding at the interface between S_1.15_ and ACFRP was the primary mode of fracture in S_1.15_/ACFRP, which significantly decreased the bending stiffness of the specimens. Importantly, the reinforcement layers of ACFRP remained undamaged during the entire loading process.
(4) The S_1.15_/ACFRP specimens exhibited lower bending properties compared to the S_1.45_ specimens. Therefore, the S_1.15_/ACFRP hybrid structure, despite being lighter, cannot effectively replace the DP780 steel sheet with a thickness of 1.45 mm in engineering applications. This was mainly due to the lower longitudinal modulus of the ACFRP hybrid composite compared to that of DP780 steel, resulting in lower bending stiffness in the initial phase of loading.
(5) Based on the results of this study and the existing literature, it can be concluded that assembling steel sheets and FRP composites by adhesive bonding may help to reduce the weight of thin-walled steel structures while maintaining their mechanical properties. However, this is only achieved when FRP is reinforced with high-performance fibers, especially those with a high longitudinal elastic modulus and high ultimate tensile strain.

## Figures and Tables

**Table 1:** An overview of steel/CFRP hybrid structures tested under bending conditions.

Ref.	Object of Study	Type of Reinforcement in CFRP	Research Area/Main Issues
[20]	B-pillar	unidirectional and bidirectional	impact test, weight reduction
[21]	B-pillar	bidirectional	drop tower test, weight reduction
[22]	B-pillar	bidirectional	impact test, weight reduction
[23]	B-pillar	unidirectional	impact test, weight reduction
[24]	B-pillar	unidirectional	drop tower test, weight reduction
[25]	specimen	bidirectional	V-bending test, bending properties
[26]	specimen	unidirectional	four-point bending test, bending properties
[27]	specimen	bidirectional	end-notched flexure test, delamination

**Table 2:** Selected mechanical properties of the DP780 grade steel.

Yield Strength (MPa)	Tensile Strength (MPa)	Elongation (%)	Ref.
546	792	23.3	[31]
530	842	18.5	[33]

**Table 3:** Specification of the hybrid aramid/carbon fiber fabric used in this study.

Weave	Areal Density (g/m^2^)	Thickness (mm)	Type and Density of Aramid Fibers	Type and Density of Carbon Fibers
plain 1/1	165 (±4%)	0.23 (±15%)	Kevlar^®^ 49 1580 dtex 1.44 g/cm^3^	Toray 3 K 200 tex 1.76 g/cm^3^

**Table 4:** Selected properties of the Biresin^®^ CR122 + CH122-5 epoxy matrix system.

Elastic Modulus (MPa)	Tensile Strength (MPa)	Elongation (%)	Density (g/cm^3^)
2800	84	5.6	1.16

**Table 5:** Measured properties of the specimens tested in this study.

Specimens	Thickness (mm)	Weight (g)	Fiber Volume Fraction in ACFRP (%)
S_1.15_/ACFRP	1.575 ± 0.017	22.745 ± 0.083	58.42 ± 2.17
S_1.15_	1.153 ± 0.004	21.517 ± 0.048	–
ACFRP	0.631 ± 0.022	1.738 ± 0.058	47.79 ± 1.46
S_1.45_	1.454 ± 0.005	27.231 ± 0.115	–

**Table 6:** Summary of the bending test results for the S_1.15_/ACFRP specimens with reference to Figure 7b.

Points on the Average Load–Deflection Curve	Average Values of Deflection δ (mm) and Load F (N) for Three Specimens	
NL (start of non-linear stage)	${\bar{\delta }}_{\mathrm{NL}}$	2.32 ± 0.11
	${\bar{F}}_{\mathrm{NL}}$	147.19 ± 14.54
DI (damage initiation)	${\bar{\delta }}_{\mathrm{DI}}$	5.41 ± 0.27
	${\bar{F}}_{\mathrm{DI}}$	264.98 ± 21.45
MAX (maximum load)	${\bar{\delta }}_{\mathrm{MAX}}$	7.45 ± 0.28
	${\bar{F}}_{\mathrm{MAX}}$	305.81 ± 4.27
UD (ultimate deflection)	${\bar{\delta }}_{\mathrm{UD}}$	9.78 ± 1.17
	${\bar{F}}_{\mathrm{UD}}$	284.35 ± 18.29
CD (complete debonding)	${\bar{\delta }}_{\mathrm{CD}}$	9.79 ± 1.17
	${\bar{F}}_{\mathrm{CD}}$	212.86 ± 1.53

**Table 7:** Average bending properties of the S_1.15_/ACFRP specimens.

$\bar{BS}$ (N/mm)	$\bar{MBLC}$$\;={\bar{F}}_{MAX}$ (N)	$\bar{AE}$ (N·mm)
63.28 ± 3.87	305.81 ± 4.27	1417.75 ± 10.83

**Table 8:** Summary of the bending test results for the S_1.45_ and S_1.15_ specimens and their average bending properties with reference to Figure 9c.

Specimen	${\bar{\delta }}_{NL}$ (mm)	${\bar{F}}_{NL}$ (N)	$\bar{F}\prime_{MAX}$$=\bar{MBLC}\prime$ (N)	$\bar{BS}$ (N/mm)	$\bar{AE}$ (N·mm)
S_1.45_	1.78 ± 0.11	171.57 ± 2.41	345.37 ± 14.45	96.43 ± 5.63	1797.34 ± 72.15
S_1.15_	1.75 ± 0.08	85.39 ± 7.17	204.19 ± 11.57	48.79 ± 2.98	1004.12 ± 52.58

**Table 9:** Summary of the bending test results for the ACFRP specimens and their average bending properties.

${\bar{\delta }}_{NL}$ (mm)	${\bar{F}}_{NL}$ (N)	$\bar{F}\prime_{MAX}$$\;=\bar{MBLC}\prime$ (N)	$\bar{BS}$ (N/mm)	$\bar{AE}\;(N\cdot \mathrm{mm})$
4.25 ± 0.28	2.23 ± 0.17	3.78 ± 0.27	0.52 ± 0.04	14.54 ± 1.10

**Table 10:** Selected approximate properties of various steel-FRP hybrid structures subjected to bending (here, BD denotes bidirectional, and UD signifies unidirectional).

Hybrid Structure	Steel Sheet	Reinforcement	Weight Increment	BS Improvement	MBLC Improvement
steel/ACFRP (in the current study)	DP780	BD aramid/carbon hybrid fabric	5.7%	30%	50%
steel/GFRP/CFRP [34]	22MnB5	UD carbon fiber mat and BD glass fabric	27.9%	368%	399%
steel/CFRP [35]	DP980	UD carbon fabric	8.2%	127%	170%
steel/CFRP [35]	DP980	UD carbon fabric	6.3%	75%	127%
steel/AFRP [35]	DP980	UD aramid fabric	11.7%	140%	234%

## Data Availability

The data presented in this study are available upon request from the author.
